# Gas-Sensor Drift Counteraction with Adaptive Active Learning for an Electronic Nose

**DOI:** 10.3390/s18114028

**Published:** 2018-11-19

**Authors:** Tao Liu, Dongqi Li, Jianjun Chen, Yanbing Chen, Tao Yang, Jianhua Cao

**Affiliations:** School of Microelectronics and Communication Engineering, Chongqing University, No. 174 Shazheng Street, Shapingba District, Chongqing 400044, China; Dongqili@cqu.edu.cn (D.L.); yanbingchen@cqu.edu.cn (Y.C.); 20181213022t@cqu.edu.cn (T.Y.); jianhuacao@cqu.edu.cn (J.C.)

**Keywords:** electronic nose, drift counteraction, active learning, online

## Abstract

Gas sensors are the key components of an electronic nose (E-nose) in violated odour analysis. Gas-sensor drift is a kind of physical change on a sensor surface once an E-nose works. The perturbation of gas-sensor responses caused by drift would deteriorate the performance of the E-nose system over time. In this study, we intend to explore a suitable approach to deal with the drift effect in an online situation. Considering that the conventional drift calibration is difficult to implement online, we use active learning (AL) to provide reliable labels for online instances. Common AL learning methods tend to select and label instances with low confidence or massive information. Although this action clarifies the ambiguity near the classification boundary, it is inadequate under the influence of gas-sensor drift. We still need the samples away from the classification plane to represent drift variations comprehensively in the entire data space. Thus, a novel drift counteraction method named AL on adaptive confidence rule (AL-ACR) is proposed to deal with online drift data dynamically. By contrast with conventional AL methods selecting instances near the classification boundary of a certain category, AL-ACR collects instances distributed evenly in different categories. This action implements on an adjustable rule according to the outputs of classifiers. Compared with other reference methods, we adopt two drift databases of E-noses to evaluate the performance of the proposed method. The experimental results indicate that the AL-ACR reaches higher accuracy than references on two E-nose databases, respectively. Furthermore, the impact of the labelling number is discussed to show the trend of performance for the AL-type methods. Additionally, we define the labelling efficiency index (LEI) to assess the contribution of certain labelling numerically. According to the results of LEI, we believe AL-ACR can achieve the best effect with the lowest cost among the AL-type methods in this work.

## 1. Introduction

An electronic nose (E-nose) is a potential approach to perform odour analysis. A typical E-nose system ordinarily consists of two parts: a gas-sensor array and a pattern-recognition unit. The former part generates odour fingerprints from gas-sensor responses with high cross sensitivity while the latter performs data analysis using computational algorithms. With this structure, E-noses can identify complicated gas components with low-cost gas-sensor arrays. Owing to the advantages in cost and operation, E-noses have been adopted in environmental monitoring [1], the food industry [2], agriculture [3] and medicine [4]. The modern E-nose system has appeared as an artificial intelligence machine since the 1990s [5]. There are several scientific fields, such as artificial intelligence, system integration and sensor technology supporting the development of E-noses.

Gas-sensor drift in E-nose systems is a kind of concept drift, which frequently occurs by surface aging, environmental disturbance and sensor poisoning. This phenomenon deteriorates the compatibility between gas-sensor responses and algorithm models over time, and finally causes the performance degradation of E-noses. In other words, the algorithm models would be meaningless without any drift calibrations over time. Thus, anti-drift methods are necessary for E-nose systems in their life-long working process. To reduce the drift effect of E-noses, possible solutions have been focused on sensor improvement [6] and algorithm modification [7]. According to current studies, algorithm-modification approaches have received increasing attention due to the achievements of artificial intelligence and machine learning. Signal pre-processing and classifier updating are two mainstreams in algorithm modification. The former manner extracts drift-like component from original gas-sensor responses and then restructures responses without drift. Huang et al. adopted principal component analysis (PCA) and common PCA (CPCA) at different gas concentrations to compensate drift signals [8]. Ziyatdinov et al. used CPCA to discover a drift direction for all gasses in feature space [9]. Besides PCA, orthogonal signal correction (OSC) [10] and independent component analysis (ICA) [11] are alternatives for drift counteraction in the time domain. Furthermore, the wavelet is another way to remove drift component in time-frequency domain [12]. In terms of classifier updating, the classifier ensemble is a popular strategy for long-term drift migration [13,14,15]. This strategy provides a weighted result based on several sub-classifiers generated from different samples. Moreover, some scholars used adaptive classifiers including self-organize maps (SOM) [16,17], adaptive resonance theory (ART) [18,19] and immune algorithm [20], to handle drift distribution adaptation dynamically. Recently, the algorithm modification manner has been extended to solve the drift problem in semi-supervised learning situations. Liu et al. put a transfer learning paradigm into a semi-supervised scene for drift elimination [21]. All the above methods have a common premise that a calibration set with complete categories and well-labelled samples should be prepared for drift correction. However, this demand would be barely satisfied in online scenarios due to inadequate time and labour to obtain such a calibration set, for example, in online toxic-gas monitoring. In this application, toxic gases rare appear while non-hazardous gases are majority. Therefore, the collected calibration set is imbalanced, which would decline the performance of the E-nose in toxic-gas recognition. Additionally, we assume that the toxic-gas recognition would be a failure according to the unreliable labels of calibration samples from the classifiers.

In this study, we use active learning (AL) to solve the calibration-set problem. AL methods can select a certain number of instances from recent incoming samples, then label the selected instances and update classifiers reliably. We believe this working process can adapt well to online scenarios due to the fact that AL methods have the ability to select relatively optimal instances from undetermined calibration samples for classifier upgrading. Moreover, conventional AL instance-selection strategies select instances with low confidence, which cannot represent the current distribution of drifting samples entirely. Consequently, we have proposed an innovative AL method on the adaptive confidence rule (AL-ACR) to balance the categories of selected instances. It is helpful to enhance the uniformity of drift information in a calibration set. We use two E-nose drift databases as benchmarks to evaluate the performance of the proposed method in drift counteraction. One dataset is public and the other was collected by the authors. According to the experimental results, we infer that the proposed AL-ACR is suitable for online drift calibration. The AL-ACR shows adequate adaptability and feasibility in online scenarios and reaches better accuracy comprehensively than other references. Considering the time-consuming and laborious requirements of manual labelling, it is crucial to obtain a higher accuracy with a lower labelling number. We therefore define the labelling efficiency index (LEI) to assess the efficiency of AL methods. The results of LEI show that AL-ACR has obvious advantages in most cases.

In this paper, we explain the development of AL in Section 2. Section 3 describes the details of the proposed AL method. In Section 4, we show and discuss the comparative results of the proposed and reference methods upon two databases. Finally, we summarize some conclusions from the experimental results in Section 5.

## 2. Related Work

The AL paradigm generally consists of two modules, a selecting engine and a learning engine [22]. The selecting engine is designed to achieve high-quality unlabelled samples according to the classifiers constructed by the learning engine. It labels the selected samples manually and adds the labelled instances to the training set. The learning engine is responsible for renewing classifiers by the both training set and the manual labelled instances, which makes the performance of the classifier improve continuously. The cycle of “selecting-learning” would be stopped if certain termination condition were reached. It is obvious that how to select high-quality instances from unlabelled samples on feature information is a key part of the AL method. It plays an important role in promoting the classification performance of AL. Therefore, studies on instance-selection strategies have gradually increased.

Membership query synthesis (MQS), stream-based and pool-based methods are typical methods proposed to select unlabelled samples. MQS is the earliest idea of AL using a query to learn [23]. In this method, the hypothetical learning system can ask questions from experts, that is, the MQS determines labels of certain instances by query. As for the instance selection of MQS, all unlabelled samples are handed to the expert for labelling, regardless of labour cost and data distribution. To avoid unnecessary labelling, scholars have proposed stream-based AL which selects instances according to either information entropy or distribution similarity and labels them in an instance-by-instance manner [24]. Although the stream-based AL has solved the drawbacks of the MQS to a certain extent, it still demands a fixed threshold to measure the information contained in each input sample. Thus, unsteady labelling density and rigid parameter adaptation would limit the performance of stream-based AL. Consequently, Lewis et al. proposed a pool-based AL method to form a “pool” for expert labelling [25]. This pool-based AL selectively labels instances that exist in a dataset to enhance the performance of classifiers. In fact, this method becomes equivalent to the stream-based AL when just one unlabelled sample exists in the pool. In other words, the stream-based AL is a special case of the pool-based one. For the instance-selection methods of pool-based AL, how to select the most representative instances from the candidates in order to label is crucial. Uncertainty sampling (US) and query-by-committee (QBC) strategies are main approaches for instance selection. The former identifies samples on distributions while the latter selects instances according to the outputs of classifiers.

Recently, in the studies of E-noses, Jiang X et al. proposed an enhanced QBC (EQBC) -radial basis function neural network (RBFNN) [26] for the recognition improvement on the QBC strategy. However, there are hardly any pubic reports for drift counteraction of E-noses with active learning. As far as we know, this study is the first research on drift compensation of E-noses with AL methods.

## 3. Methods

### 3.1. Baseline Methods

There are three AL methods including US, QBC and EQBC presented as baseline for comparison. Considering the various variants of them, we use US proposed in Ref. [27], QBC and EQBC in Ref. [26].

The US strategy uses the classifiers to identify the largest indeterminacy of unlabelled samples for subsequent labelling. The focus of US is to measure the uncertainty of the sample. It assumes the most uncertain sample contributes to the improvement of classification at most. We can obtain the optimal sample for recognition improvement by calculating posterior probability. We compute the *margin_i_* of instance *p_i_* as follow:

(1) $$margin_{i}=f^{E}({\hat{y}}_{c1}|p_{i})-f^{E}({\hat{y}}_{c2}|p_{i})$$

where ${\hat{y}}_{c1}$ and ${\hat{y}}_{c2}$ are, respectively, the class with the maximum posteriori probability and the second most posteriori probability. The margin-based metric is prone to select instances with minimum *margin_i_* between posteriori probabilities of the two most likely class labels.

The QBC method is the most popular AL method based on version space reduction. It aims to select samples in a simplified space for man-made labelling, which has been proposed by Seung et al. [28] and Freund et al. [29]. The QBC sets up a committee group to vote a sample with the highest disagreement for expert labelling. We choose Kullback–Leibler (KL) [30] as the metric for QBC to measure the valuable instances for classification promotion. The calculation formula of KL can be given by Equations (1) and (2) as follows:

(2) $$e_{i}=\frac{1}{K}\sum_{k=1}^{K}D(P_{k}(C|p_{i})||\frac{\sum_{k}P_{k}(C|p_{i})}{K})\;$$

(3) $$D(P_{k}(C|p_{i})||\frac{\sum_{k}P_{k}(C|p_{i})}{K}=\sum_{m=1}^{\left|C\right|}P_{k}(c_{m}|p_{i})\log\frac{K\cdot P_{k}(C|p_{i})}{\sum_{k}P_{k}(C|p_{i})}\;$$

where $P_{k}(c_{m}|p_{m})$ represents the probability that sample *p_i_* is labelled as Class *c_m_* by member *k* and *K* denotes the total number of committee members.

The EQBC method is based on a weighted combination of Kullback–Leibler divergence (KL−d) $e_{i}{}^{KL-d}$ and vote entropy (VE) $e_{i}{}^{VE}$ as shown in Equation (4).

(4) $$e_{i}=\omega_{1}e_{i}{}^{KL-d}+\omega_{2}e_{i}{}^{VE}\;$$

where *ω*_1_ and *ω*_2_ are two adjustable weighted parameters.

### 3.2. Adaptive Instance Selection

Baseline AL methods regularly select useful instances with a single criterion with either high information entropy or low similarity, which is suitable to static data due to the constant data distribution. The classification plane is more distinguished by the increasing number of selected instances, regardless of the instance expiration. Unfortunately, in online mode, instance expiration is bound to exist under drift influence. This limitation may lead AL methods based on single criterion to barely reflect current drift trend by category in time. Finally, in a period, the performance of one class may rise, whereas others would incline. Thus, we proposed an innovative pool-based AL method, AL-ACR, with different instance-selection criteria adaptively. There are two criteria used here to discover the instances evenly in distribution by category. We adopt information entropy to index the necessity of samples for labelling. A high information-entropy value shows low confidence, vice versa.

**Figure 1:**
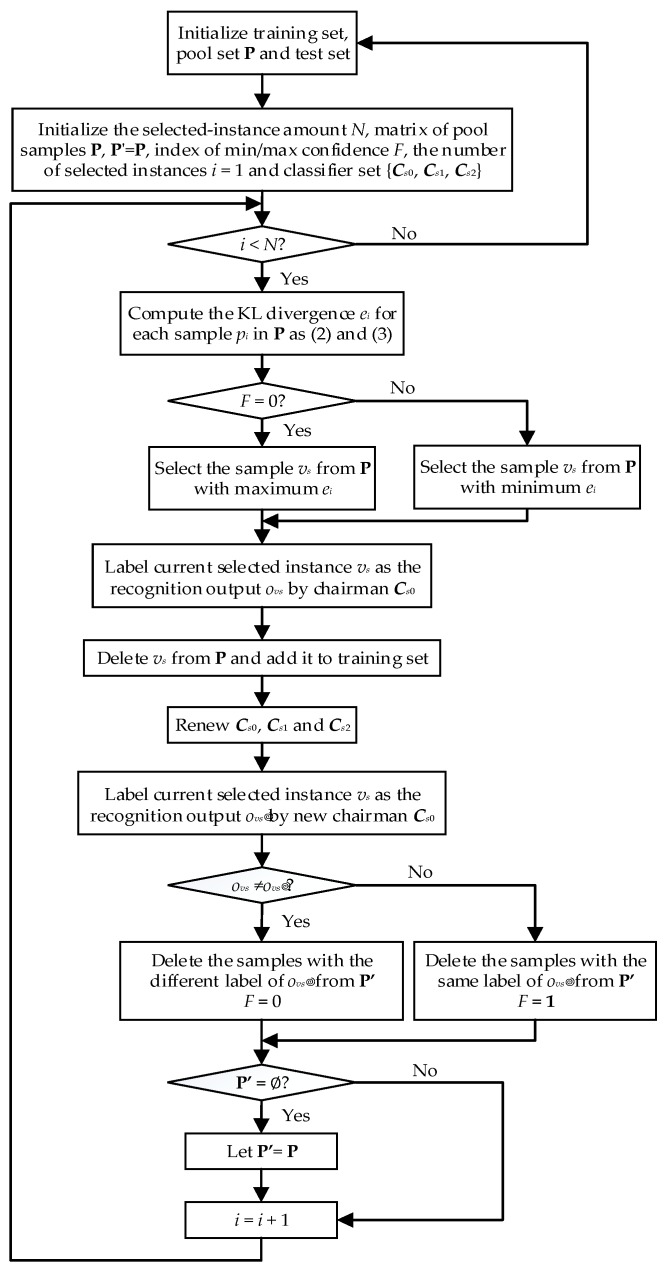
Flow chart of active learning on adaptive confidence rule (AL-ACR) algorithm.

As Figure 1 described, the AL-ACR method implements instance selection and classifier promotion iteratively. *F* is the index of min/max confidence and its default value equals 0. *F* = “0” denotes that the minimum confidence should be used while *F* = “1” infers that the maximum confidence should be selected. For classifiers, the chairman classifier ***C****_s_*_0_ is trained by the whole training set, and the member classifiers ***C****_s_*_1_ and ***C****_s_*_2_ are generated from parts of the training set randomly. In an instance-selection process, we primarily compare the chairman outputs of the current instance before and after classifier updating. On the one hand, if they are different, it means the classification plane of the chairman needs to be distinguished, then the sample with maximum information entropy to specify the classification boundary should be selected in the next round. On the other hand, if they are the same, we assume the category of current instance is well classified, then instances belonging to other kinds should be designated with minimum information entropy according to label diversity and distribution equilibrium. For classification promotion, when entering a new instance, the corresponding label is assigned by expert opinion. After that, both the new instance and corresponding label are added to the training sets belonging to the members of the committee. Next, the members are respectively retrained by their training sets.

## 4. Experiments and Results

### 4.1. Datasets

#### 4.1.1. Public Dataset

The public dataset (http://archive.ics.uci.edu/ml/machine-learning-databases/00224/) is from the machine learning repository of University of California Irvine [31]. We denote this dataset as Dataset A for short in following discussion.

**Figure 2:**
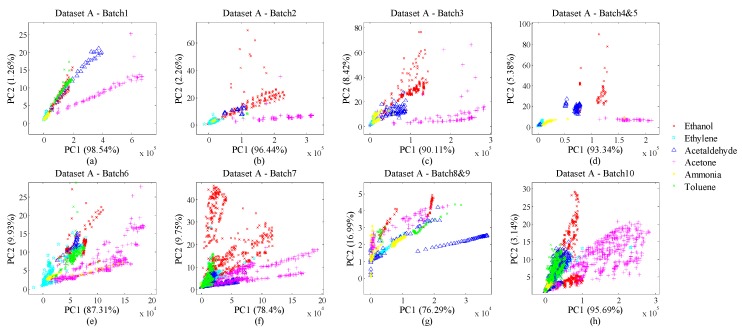
(**a**) Data distribution of Batch 1; (**b**) Data distribution of Batch 2; (**c**) Data distribution of Batch 3; (**d**) Data distribution of Batch 4&5; (**e**) Data distribution of Batch 6; (**f**) Data distribution of Batch 7; (**g**) Data distribution of Batch 8&9; (**h**) Data distribution of Batch 10.

There are totally 13,910 samples collected by an E-nose over three years (36 months). The gas-sensor array consists of sensor devices (4 of each) as TGS2600, TGS2602, TGS2610 and TGS2620; in total 16 gas sensors are adopted in the E-nose system. This dataset includes six-gas samples dosed at different concentrations. The entire response of a gas sensor in an experiment can be divided into three phases: injection, measurement and sweeping. Consequently, 8 features (2 steady-state features and 6 transient features) have been abstracted from these three phases for each sensor. On account of the sensor array made up of 16 gas sensors, one experiment can collect 128 (8 × 16) features. Thus, the total size of Dataset A is 13,910 × 128. All 10 batches have been arranged by time order. To reduce the imbalance of batch sizes, we integrated Batch 4 and 5 as Batch 4&5, Batch 8 and 9 as Batch 8&9. Thus, we actually prepared 8 bunches of drifting samples for evaluation. Additionally, the working temperature of the gas sensors was maintained at 400 °C and the sampling rate was set to 100 Hz. Figure 2a–h shows the distributions of the 8-bunch samples in principal component analysis (PCA) plots. We can infer that the drift effect acts apparently on the samples in all batches. The distribution of data varies continuously with time, especially between different batches. We believe that classifiers worked with no drift counteraction would be invalid rapidly.

#### 4.1.2. Collected Dataset

**Figure 3:**
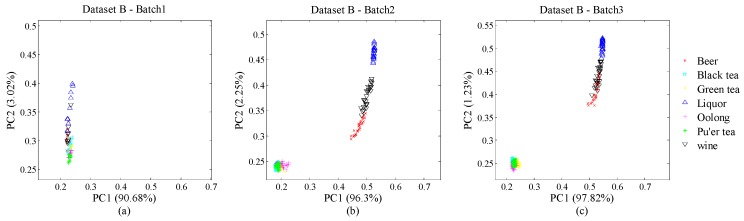
(**a**) Data distribution of Batch 1 of Dataset B; (**b**) Data distribution of Batch 2 of Dataset B; (**c**) Data distribution of Batch 3 of Dataset B.

Besides Data A, the other dataset was collected from an E-nose system we designed. This E-nose system consists of three parts: gas sensor array, control unit and upper computer. The gas-sensor array includes 32 gas sensors with solid electrolyte, electrochemical and metal oxide types. The control unit transfers the fingerprints from the gas-sensor array to the upper computer for further processing. We gathered 63, 189 and 189 samples in the first, third and fourth months, respectively. Consequently, we divided the 4-month dataset into three batches according to chronological order and each batch had all kinds of samples equally in quantity. For each sample, we used the steady-state value of certain gas-sensor responses as a feature. Thus, one gas sensor corresponds one feature and one sample can be denoted as a 32-dimensional vector according to 32 gas sensors. Here, we define this dataset as Dataset B for following discussions. Figure 3a–c illustrate the distribution of Dataset B with PC1 and PC2 by batch.

### 4.2. Experimental Setup

Our evaluation contains four parts. We primarily evaluated the accuracy of proposed AL-ACR compared with those of other start-of-the-art methods (AL-US, AL-QBC, AL-EQBC, RBF-SVM, GFK-SVM, Comgfk-SVM, Comgfk-ML and RBF-ML [21]) in long-term online drift counteraction. Secondly, we defined 4 different long-term scenarios to assess the performance between AL methods with labelled-instance number, category balance and labelling efficiency. The defined Scenarios 1–4 are as follows:

- Scenario 1 (Dataset A): Batch 1 for initial training, Batch 2–10 for online testing.
- Scenario 2 (Dataset A): Batch 2 for initial training, Batch 3–10 for online testing.
- Scenario 3 (Dataset A): Batch 3 for initial training, Batch 4–10 for online testing.
- Scenario 4 (Dataset B): Batch 1 for initial training, Batch 2 and 3 for online testing.

For each scenario, data pre-processing has been performed to normalize sample features in [0, 1] by:

(5) $$\tilde{x}=\frac{x}{x_{\max}+x_{\min}}\;$$

where x represents a 128-dimensional sample, $\tilde{x}$ is the output after pre-processing, $x_{\max}$ and $x_{\min}$ is the maximum and minimum feature value in **x** respectively.

In terms of classifiers, we adopt three types: *k*-nearest neighbour (*k-*NN), support vector machine (SVM) and radial basis function neural network (RBFNN). The parameter *k* of *k-*NN is set to 3. For SVM, we adjust kernel function, kernel parameter *σ* (kernel function only) and penalty factor *C* to optimize the performance of SVM. During optimization of SVM, either radial basis or linear function could be selected. We set the scope of kernel parameter *σ* and the finest value of *C* to the range 10^−3^ to 10^3^ with variable step. Considering the computational complexity and overall performance, we finally choose linear function as our option and set *C* = 0.2 (dataset A) or *C* = 2 (Dataset B). For RBFNN, we set the number of hidden layers to 50, the training error to 10^−5^ and the variance of radial basis kernel to 1.

For AL methods, we chose roughly 30% of the data as pool samples per batch while the remains are used for testing only. We consider that it is impossible to label instances at any time in reality. Hence, the time of labelling should be concentrated in a period per batch. In the following section, on account of the fairness, the accuracies of the AL methods are obtained on whole batches including both pool and testing data in Table 1. Others are concluded on the testing data due to the comparison just between AL methods.

### 4.3. Results and Discussion

#### 4.3.1. Performance Evaluation of Paradigms

Table 1 has shown the accuracies of different paradigms in the Scenario 1. The supervised paradigm methods, RBF-SVM, GFK-SVM, Comgfk-SVM, are SVM with radial basis, geodesic flow and associated combined kernel, respectively. Another paradigm is semi-supervised learning including Comgfk-ML and RBF-ML. In terms of the AL paradigm, AL-US, AL-QBC, AL-EQBC and the proposed AL-ACR methods are used for comparison. We abstract the results of RBF-SVM, GFK-SVM, Comgfk-SVM, Comgfk-ML and RBF-ML directly from Ref. [21]. In this subsection, we calculate the results of AL methods in scenario 1 and use SVM only for performance comparison owing to the same settings adopted in Ref. [21].

We set the number of selected instance *N* to 6 for AL methods, a comparatively small number leading to the indistinctness of drift description. Reasonably, the performance of AL methods would be restricted to a poor level in the range of *N*. Among all the methods with different paradigms, AL-ACR has clearly reached the best recognition performance. The above phenomena indicate that the proposed method is suitable for online drift suppression in most cases. For the paradigm, we believe the supervised learning manner cannot compensate drift well for a long time; the semi-supervised learning methods are weak in robustness due to the unreliability of online labels.

#### 4.3.2. Effect of Instance Number

The number of labelled instances *N* for each batch is an important parameter of AL methods. We investigate the recognition-rate shift of the AL-ACR models by increasing *N* value from small to large. In order to avoid class imbalance by the number of labelled instances, we set the increasing step of *N* to the category size of the dataset. Thus, for Scenarios 1–3, the step of *N* is 6 while that of Scenario 4 is 7.

**Figure 4:**
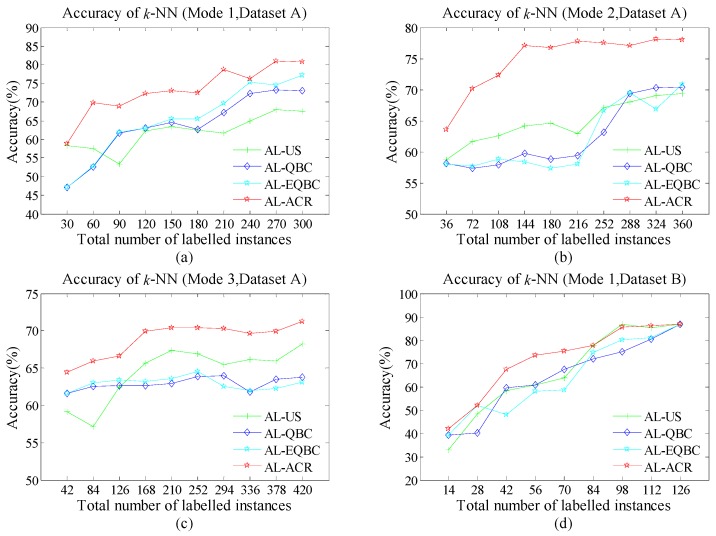
(**a**) Accuracy of *k*-nearest neighbour (*k*-NN) (Scenario 1); (**b**) Accuracy of *k*-NN (Scenario 2); (**c**) Accuracy of *k*-NN (Scenario 3); (**d**) Accuracy of *k*-NN (Scenario 4).

**Figure 5:**
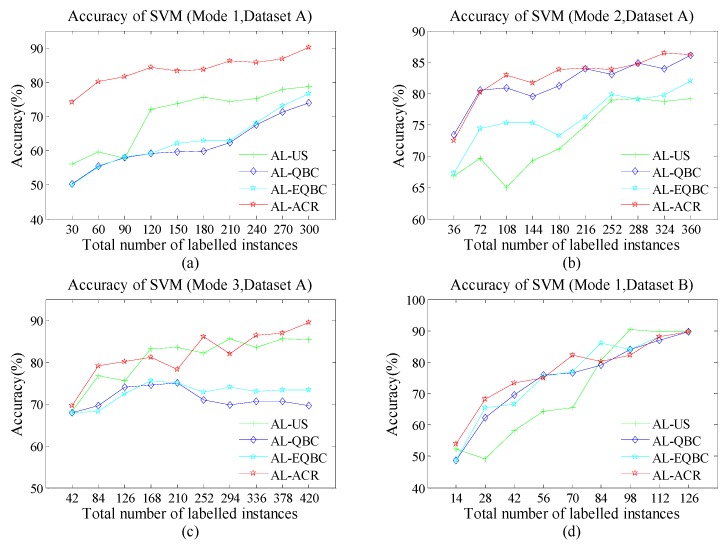
(**a**) Accuracy of support vector machine (SVM) (Scenario 1); (**b**) Accuracy of SVM (Scenario 2); (**c**) Accuracy of SVM (Scenario 3); (**d**) Accuracy of SVM (Scenario 4).

**Figure 6:**
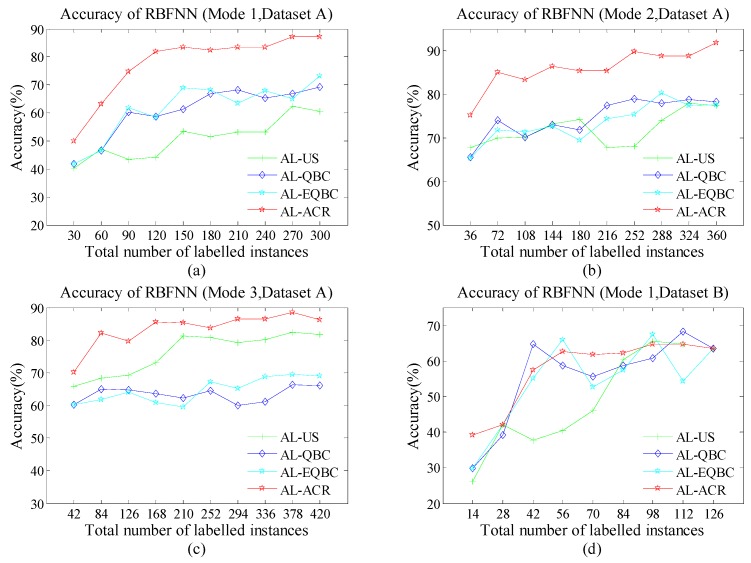
(**a**) Accuracy of radial basis function neural network (RBFNN) (Scenario 1); (**b**) Accuracy of RBFNN (Scenario 2); (**c**) Accuracy of RBFNN (Scenario 3); (**d**) Accuracy of RBFNN (Scenario 4).

As Figure 4a–d, Figure 5a–d and Figure 6a–d show, the green line with cross, indigo line with diamond, blue line with star and red line with star represent the accuracy of AL-US, AL-QBC, AL-EQBC and AL-ACR, respectively. The recognition rates of three AL methods are presented. We can infer that larger *N* causes higher recognition rate in all cases. We believe the drift information has been described more abundantly and accurately since *N* increased. Compared with the other three methods, AL-ACR has achieved the best accuracy in most cases. This confirms the significant effectiveness of AL-ACR for online drift suppression of E-noses once again and the performance of reference methods is lagging behind AL-ACR. In other words, AL-ACR is an optimal choice almost under different parameters *N* from the results of Figure 4a–d, Figure 5a–d and Figure 6a–d.

For both datasets A and B, the performance of algorithms has entered the bottleneck or even a certain decline since *N* became large. This is reasonable due to drift-information redundancy caused by the mismatch between *N* and the drift-information time scale. Such redundant information is meaningless to the AL models, and even lead to negative effects.

Additionally, we use three classifiers, *k-*NN, SVM and RBFNN, to test whether the performance of the proposed methods has selectivity for classifiers. We note that all the classifiers share almost the same trend in terms of accuracy. On the one hand, SVM often shows stronger recognition ability than *k-*NN and RBFNN. This may be caused by the fact that SVM searches for optimal solutions globally whereas *k-*NN is based on local discriminate and RBFNN may suffer over-fitting. On the other hand, the performance difference of all four methods seems smaller on SVM than both *k*-NN and RBFNN. We infer that the excellent recognition ability of SVM may have offset the drawbacks of AL-US, AL-QBC and AL-EQBC.

#### 4.3.3. Distribution of Labeled Instances

As previously described, the AL-ACR method tries to select instances evenly by category. This action would help the classifiers to capture entire drift details near and away from the classification boundaries during online drift compensation.

**Figure 7:**
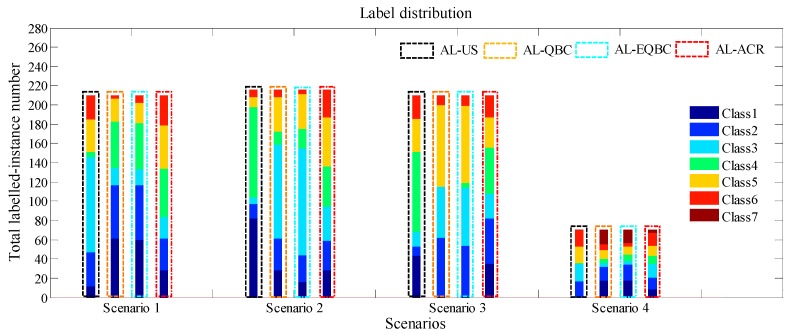
Label distribution in Scenarios 1–4.

As Figure 7 and Table 2 show, we present the true labels of selected instances with different colours in all scenarios. Black, ochre, blue and red dotted box denotes the bar of AL-US, AL-QBC, AL-EQBC and AL-ACR, respectively. The height of the bar denotes the instance quantity of a certain category. This allows us to see 4 bars in each scenario. Furthermore, we set total number of labelled instances to 210, 216, 210 and 70 for Scenarios 1–4 respectively. We can infer from the results that AL-ACR has the most balanced instance category to achieve a complete drift trend in online mode. The other three methods are slightly worse than AL-ACR in label balance. According to the ratios of instance classes in Table 2, we can find more balanced category distribution appeared in AL-ACR, which can properly explain why AL-US, AL-QBC and AL-EQBC perform weaker in terms of accuracy.

#### 4.3.4. Values of Labelling Efficiency Index (LEI)

In general, we hope the AL methods can not only achieve high accuracy, but also reduce the number of labels to save cost in terms of time and laboriousness. In other words, raising the accuracy increment per labelling is crucial in AL. Therefore, we define LEI to demonstrate the cost of a certain AL method in recognition improvement. The LEI is a hybrid index as follows:

(6) LEI=α⋅Acc+(1−α)⋅ΔAcc 

where *Acc* is the accuracy under the number of selected instances *N*, Δ*Acc* is the increment of accuracy since the last labelling and *α* is a adjust parameter belongs to [0, 1]. We define LEI as a sum of two terms that represent current performance and associated increment, respectively. A qualified AL method should have excellent classification ability and economic cost of performance enhancement simultaneously. We set *α* to 0.2 for LEI computation.

Table 3, Table 4, Table 5, Table 6, Table 7 and Table 8 illustrate the LEI values in all scenarios on the three classifiers with different *N*. In Scenario 1, no matter either SVM, *k*-NN or RBFNN, AL-ACR reaches the highest LEI score from beginning to end. In Scenario 2, AL-ACR is still the winner in all cases on both *k*-NN and RBFNN while 7 out of 10 cases on SVM are best with AL-ACR. As for Scenarios 3 and 4, although the performance of AL-ACR sometimes falls down compared with other references, it still keeps the highest LEI values in most cases. In a word, the AL-ACR has the higher labelling efficiency compared with AL-US, AL-QBC and AL-EQBC in different scenarios almost. We assume that AL-ACR is a very economical and practical approach to use for drift counteraction of E-noses in online mode.

## 5. Conclusions

To solve the online drift problem, we redefine a novel AL method, namely AL-ACR, to overcome the imbalance problem of drift information. Instance selection is performed with adaptive rules. Experimental results prove that the proposed method overmatches other references in online working. Furthermore, AL-ACR has obvious advantages in recognition, parameter sensitivity, instance equilibrium and labelling efficiency. It is a favorable choice for the online drift counteraction of E-noses. Future work should be focused on the online mechanism of pool refreshing and online AL implementation in a limited storage resource.

## Figures and Tables

**Table 1:** Accuracy of methods in Scenario 1 (%).

	RBF-SVM	GFK-SVM	Comgfk-SVM	Comgfk-ML	RBF-ML	AL-US	AL-QBC	AL-EQBC	AL-ACR
Batch 2	74.36	72.75	74.47	80.25	42.25	53.55	65.34	65.23	**98.61**
Batch 3	61.03	70.08	70.15	74.99	73.69	83.26	65.83	65.80	**87.39**
Batch 4&5	32.96	68.64	68.20	72.53	70.70	57.93	69.76	69.76	**72.64**
Batch 6	28.26	73.82	73.99	77.82	77.51	51.62	44.18	44.18	**93.12**
Batch 7	28.81	54.53	54.59	**71.68**	54.43	60.08	42.38	42.38	69.52
Batch 8&9	28.80	64.16	**64.71**	50.47	27.39	66.24	40.96	40.96	55.76
Batch 10	34.47	41.78	53.79	53.79	34.92	59.88	37.95	37.93	**55.72**
Average	36.09	55.72	57.49	60.19	47.61	54.07	45.80	45.78	**66.60**

**Table 2:** Category distribution in Scenarios 1–4 (selected number/ratio).

		Class 1	Class 2	Class 3	Class 4	Class 5	Class 6	Class 7
SCENARIO 1	**US**	12/5.71%	35/16.67%	99/47.14%	5/2.38%	34/16.19%	25/11.90%	0/0.00%
	**QBC**	61/28.24%	56/25.93%	18/8.33%	48/22.22%	24/11.11%	3/1.39%	0/0.00%
	**EQBC**	60/28.57%	57/27.14%	16/7.62%	48/22.86%	21/10.00%	8/3.81%	0/0.00%
	**ACR**	28/13.33%	33/15.71%	23/10.95%	50/23.81%	45/21.43%	31/14.76%	0/0.00%
SCENARIO 2	**US**	82/37.96%	15/6.95%	7/3.24%	94/43.52%	10/4.63%	8/3.70%	0/0.00%
	**QBC**	28/12.96%	33/15.28%	98/45.37%	13/6.02%	36/16.67%	8/3.70%	0/0.00%
	**EQBC**	16/7.41%	28/12.96%	111/51.39%	20/9.26%	36/16.67%	5/2.31%	0/0.00%
	**ACR**	28/12.96%	31/14.35%	35/16.20%	42/19.45%	51/23.61%	29/13.43%	0/0.00%
SCENARIO 3	**US**	43/20.48%	10/4.76%	15/7.14%	83/39.52%	35/16.67%	24/11.43%	0/0.00%
	**QBC**	1/0.46%	61/28.24%	53/24.54%	0/0.00%	85/39.35%	10/4.63%	0/0.00%
	**EQBC**	1/0.48%	53/25.24%	60/28.57%	5/2.38%	80/38.10%	11/5.23%	0/0.00%
	**ACR**	35/16.67%	47/22.38%	26/12.38%	48/22.86%	31/14.76%	23/10.95%	0/0.00%
SCENARIO 4	**US**	0/0.00%	17/24.29%	18/25.71%	1/1.42%	17/24.29%	17/24.29%	0/0.00%
	**QBC**	18/25.71%	14/20.00%	4/5.71%	4/5.71%	9/12.86%	6/8.57%	15/21.44
	**EQBC**	18/25.71%	16/22.86%	4/5.71%	7/10.00%	8/11.43%	4/5.71%	13/18.58%
	**ACR**	9/12.86%	12/17.14%	14/20.00%	8/11.43%	11/15.71%	13/18.57%	3/4.29%

US: uncertainty sampling; QBC: query-by-committee; EQBC: enhanced QBC; ACR: adaptive confidence rule.

**Table 3:** Labelling efficiency index (LEI) (*k*-NN) in Scenario 1–3 (%).

		N = 6	N = 12	N = 18	N = 24	N = 30	N = 36	N = 42	N = 48	N = 54	N = 60
SCENARIO 1	**US**	14.29	12.78	11.32	13.23	13.33	13.01	12.76	13.42	14.00	13.91
	**QBC**	10.54	11.45	13.37	13.40	13.60	13.04	13.96	15.01	15.17	15.09
	**EQBC**	10.54	11.52	13.39	13.40	13.80	13.68	14.53	15.68	15.43	15.94
	**ACR**	**14.46**	**16.05**	**15.12**	**15.59**	**15.55**	**15.25**	**16.51**	**15.89**	**16.84**	**16.74**
SCENARIO 2	**US**	11.85	12.57	12.71	13.05	13.09	12.68	13.61	13.78	13.98	14.03
	**QBC**	11.67	11.43	11.59	12.00	11.79	11.91	12.72	14.06	14.23	14.25
	**EQBC**	11.61	11.52	11.80	11.69	11.45	11.60	13.49	14.10	13.50	14.35
	**ACR**	**13.48**	**14.85**	**15.12**	**16.06**	**15.86**	**16.00**	**15.88**	**15.74**	**15.92**	**15.86**
SCENARIO 3	**US**	11.48	11.12	12.52	13.27	13.62	13.50	13.18	13.30	13.24	13.74
	**QBC**	12.30	12.57	12.57	12.55	12.62	12.82	12.83	12.36	12.73	12.78
	**EQBC**	12.30	12.68	12.76	12.70	12.76	12.97	12.52	12.40	12.45	12.64
	**ACR**	**13.26**	**13.46**	**13.55**	**14.25**	**14.31**	**14.26**	**14.23**	**14.07**	**14.10**	**14.38**

**Table 4:** LEI (*k*-NN) in Scenario 4 (%).

		N = 7	N = 14	N = 21	N = 28	N = 35	N = 42	N = 49	N = 56	N = 63
SCENARIO 4	**US**	7.13	10.84	12.82	13.07	13.59	16.50	**18.34**	17.88	18.13
	**QBC**	9.13	8.70	13.10	13.07	14.39	15.20	15.76	16.86	18.13
	**EQBC**	9.13	11.76	10.36	12.44	12.44	15.80	16.88	16.94	18.13
	**ACR**	**10.00**	**11.76**	**14.99**	**15.98**	**16.16**	**16.50**	18.08	**18.05**	**18.13**

**Table 5:** LEI (SVM) in Scenarios 1–3 (%).

		N = 6	N = 12	N = 18	N = 24	N = 30	N = 36	N = 42	N = 48	N = 54	N = 60
SCENARIO 1	**US**	12.96	13.02	12.22	15.40	15.57	15.84	15.46	15.57	16.08	16.22
	**QBC**	10.99	11.92	12.24	12.39	12.36	12.36	12.85	13.93	14.68	15.22
	**EQBC**	10.96	11.89	12.28	12.39	12.92	13.02	12.95	14.04	15.08	15.78
	**ACR**	**18.98**	**18.48**	**18.02**	**18.22**	**17.73**	**17.63**	**18.07**	**17.88**	**18.00**	**18.64**
SCENARIO 2	**US**	12.96	13.93	12.79	13.86	14.25	15.09	15.97	15.99	15.88	15.96
	**QBC**	**15.15**	**16.81**	16.67	16.23	16.54	17.11	16.85	**17.22**	17.00	17.42
	**EQBC**	13.13	15.19	15.30	15.25	14.75	15.38	16.17	15.97	16.09	16.56
	**ACR**	14.85	16.73	**17.17**	**16.73**	**17.15**	**17.14**	**17.03**	17.21	**17.53**	**17.46**
SCENARIO 3	**US**	13.83	16.00	15.52	**17.18**	**17.14**	16.79	**17.48**	16.98	17.39	17.32
	**QBC**	13.71	14.13	15.14	15.15	15.22	14.30	14.00	14.19	14.17	13.94
	**EQBC**	13.71	13.76	14.77	15.42	15.22	14.69	14.94	14.72	14.76	14.77
	**ACR**	**14.29**	**16.66**	**16.62**	16.71	15.95	**17.64**	16.68	**17.60**	**17.69**	**18.20**

**Table 6:** LEI (SVM) in Scenario 4 (%).

		N = 7	N = 14	N = 21	N = 28	N = 35	N = 42	N = 49	N = 56	N = 63
SCENARIO 4	**US**	15.65	12.24	13.62	14.49	14.43	17.51	19.46	19.12	18.98
	**QBC**	14.52	15.61	16.26	**17.12**	16.90	17.16	18.08	18.52	18.98
	**EQBC**	**16.15**	**17.14**	**17.21**	16.94	**18.14**	17.42	17.65	18.78	18.98
	**ACR**	15.65	12.24	13.62	14.49	14.43	**17.51**	**19.46**	**19.12**	**18.98**

**Table 7:** LEI (RBFNN) in Scenarios 1–3 (%).

		N = 6	N = 12	N = 18	N = 24	N = 30	N = 36	N = 42	N = 48	N = 54	N = 60
SCENARIO 1	**US**	9.05	10.36	9.16	9.20	11.27	10.77	11.05	11.00	12.91	12.49
	**QBC**	9.62	10.27	13.30	12.61	13.05	14.14	14.32	13.60	13.90	14.33
	**EQBC**	9.62	10.25	13.67	12.53	14.80	14.41	13.31	14.17	13.51	15.19
	**ACR**	**12.37**	**14.68**	**16.87**	**18.03**	**18.08**	**17.59**	**17.68**	**17.57**	**18.25**	**18.15**
SCENARIO 2	**US**	14.39	14.56	14.40	15.03	15.18	13.68	13.73	15.03	15.83	15.68
	**QBC**	13.65	15.62	14.40	14.99	14.64	15.82	16.11	15.88	16.03	15.87
	**EQBC**	13.57	15.02	14.69	14.88	14.08	15.15	15.33	16.38	15.72	15.73
	**ACR**	**16.86**	**18.57**	**17.63**	**18.12**	**17.70**	**17.63**	**18.49**	**18.22**	**18.14**	**18.79**
SCENARIO 3	**US**	14.70	14.61	14.53	15.23	16.95	16.77	16.33	16.45	16.90	16.71
	**QBC**	12.81	13.68	13.41	13.04	12.66	13.15	12.11	12.35	13.44	13.38
	**EQBC**	12.81	12.85	13.26	12.39	12.06	13.76	13.27	14.03	14.12	13.99
	**ACR**	**16.12**	**18.28**	**17.07**	**18.18**	**17.92**	**17.43**	**17.89**	**17.82**	**18.20**	**17.67**

**Table 8:** LEI (RBFNN) in Scenario 4 (%).

			N = 7		N = 14		N = 21		N = 28		N = 35		N = 42		N = 49	N = 56	N = 63
SCENARIO 4	**US**	8.23		10.82		8.98		9.25		10.26		13.21		14.16		13.86	13.50
	**QBC**	9.35		10.10		**15.40**		13.42		12.38		12.86		13.13		**14.63**	13.50
	**EQBC**	9.35		10.82		13.13		**15.06**		11.76		12.60		**14.59**		11.65	13.50
	**ACR**	**12.35**		**10.82**		13.70		14.33		**13.80**		**13.65**		13.99		13.86	**13.50**
